# Transgene therapy for rat anti-Thy1.1 glomerulonephritis via mesangial cell vector with a polyethylenimine/decorin nanocomplex 

**DOI:** 10.1186/1556-276X-7-451

**Published:** 2012-08-09

**Authors:** Jian-Yong Sun, Yu Sun, Hui-Juan Wu, Hong-Xia Zhang, Zhong-Hua Zhao, Qi Chen, Zhi-Gang Zhang

**Affiliations:** 1 Department of Pathology, Shanghai Medical College, Fudan University, Shanghai, China; 2 Key Laboratory of Molecular Medicine, Ministry of Education of China, Shanghai Medical College, Fudan University, Shanghai, China

**Keywords:** Polyethylenimine, Decorin, Nanocomplex, Mesangial cell, Rat anti-Thy1.1 nephritis

## Abstract

Polyethylenimine (PEI), a cationic polymer, is one of the most efficient non-viral vectors for transgene therapy. Decorin (DCN), a leucine-rich proteoglycan secreted by glomerular mesangial cells (MC), is a promising anti-fibrotic agent for the treatment of glomerulonephritis. In this study, we used PEI–DCN nanocomplexes with different N/P ratios to transfect MC in vitro and deliver the MC vector with PEI–DCN expressing into rat anti-Thy1.1 nephritis kidney tissue via injection into the left renal artery in vivo. The PEI–plasmid DNA complex at N/P 20 had the highest level of transfection efficiency and the lowest level of cytotoxicity in cultured MC. Following injection, the ex vivo gene was transferred successfully into the glomeruli of the rat anti-Thy1.1 nephritis model by the MC vector with the PEI–DCN complex. The exogenous MC with DCN expression was located mainly in the mesangium and the glomerular capillary. Over-expression of DCN in diseased glomeruli could result in the inhibition of collagen IV deposition and MC proliferation. The pathological changes of rat nephritis were alleviated following injection of the vector. These findings demonstrate that the DCN gene delivered by the PEI–DNA nanocomplex with the MC vector is a promising therapeutic method for the treatment of glomerulonephritis.

## Background

Gene therapy is a promising strategy for treatment of inheritable or acquired diseases [1-3]. One of the key factors for successful gene therapy is the development of safe and efficient gene delivery vectors. Accumulating data show that one of the most common using non-viral vectors in vivo and in vitro is polyethylenimine (PEI) [4,5]. PEI is a highly water-soluble cationic polymer in which every two carbon atoms are followed by a nitrogen atom. Under physiological conditions, approximately 20% of the nitrogens of PEI are protonated and, as a result, the ionization state of the polymer can be changed over a broad pH range [6]. The positive charge of PEI results in effective binding to the negatively charged plasmid DNA (pDNA), forming a nanoparticle of the pDNA complex that is small enough to be transported easily through the cytoplasm and to protect the pDNA from nuclease degradation [7,8]. PEI has two structural forms, linear and branched, with various molecular masses. Some studies have shown that 25 kDa branched PEI can be used as a gene vector that has many advantages, such as high transfection efficiency, low cytotoxicity, no immune reaction and easy modification in vitro [9-11]. PEI has been used successfully in many experiments and clinical trials [4,12]. For example, the intraperitoneal injection route of administration of PEI–DNA is considered to be promising for the treatment of a variety of diseases, including carcinoma of the stomach, pancreas and ovary [13,14]. Whether by aerosol inhalation or by the intranasal route, the PEI–DNA complex is potentially useful as a therapeutic approach for many lung diseases, such as cancer and asthma [15]. Delivery of the p53 tumor suppressor gene and PEI complex by aerosol can inhibit lung metastasis induced by a melanoma [16]. When administered by the intranasal route, the IL-4 antisense oligodeoxynucleotides–PEI complexes are capable of inhibiting IL-4 secretion in a sensitized murine model of airway inflammation, which is thought to be an effective therapy for asthma [17].

Glomerulosclerosis, the end stage of various progressive glomerular diseases, is characterized by over-expression of TGF-β1 and extracellular matrix deposition. Decorin (DCN) is a leucine-rich proteoglycan secreted by glomerular mesangial cells (MC) in kidney. DCN can bind to TGF-β and neutralize its biological activity, inhibit the matrix deposition in glomeruli induced by over-expression of TGF-β1 and, thus, inhibit the development of glomerulosclerosis. Earlier, we showed that the over-expression of DCN could inhibit MC growth, promote cell apoptosis, and down-regulate the protein levels of TGF-β1 and collagen type IV in vitro [18]. Using the hemagglutinating virus of Japan; Sendao virus (HVJ)-liposome method to transfer a DCN plasmid vector into rat skeletal muscle, Isaka and colleagues found increased amounts of DCN protein in skeletal muscle and in kidney with a marked therapeutic effect on rat glomerulonephritis [19]. Therefore, DCN is a promising anti-fibrotic agent for the treatment of glomerulonephritis.

Successful gene therapy is dependent on the choice of a sensitive gene and a suitable gene vector, and identification of the correct target. Therefore, it is essential in gene therapy research to explore the way of directional gene delivery to target organ. In a study of glomerular disease, it is found that the exogenous mesangial cell transferred into kidney by renal artery injection has a feature of “homing instinct” and can migrate to the mesangium of glomeruli. Kim et al. transferred rat MC with β-galactosidase expressing plasmid as a cell vector into a rat left kidney. Cells expressing enzyme activity were located in the glomerular capillary and mesangium in 80% of the glomeruli 1 day later [20]. This study provides evidence that transfer of an ex vivo gene to glomeruli is feasible and might prove to be a useful tool for future investigation of glomerular diseases. We thus used the MC cell vector to deliver a PEI–DCN expression plasmid into an animal nephritis model for gene therapy research.

In this study, we used a PEI**–**DCN nanocomplex as a gene vector to transfect cultured mesangial cells and investigated the effect of DCN in vitro. Subsequently, we transferred the PEI**–**DCN expressing MC into the left kidney of a rat anti-Thy1.1 glomerulonephritis model via left renal artery injection and assessed the feasibility of gene therapy delivered by a PEI nanocomplex in glomerular diseases.

## Methods

Polyethylenimine (PEI, branched, 25 kDa) and the monoclonal antibody to β-actin were purchased from Sigma-Aldrich (St. Louis, MO). The monoclonal anti-DCN antibody was from R&D Systems (Minneapolis, MN). The monoclonal anti-vimentin antibody was from Chamngdao Biotechnology (Shanghai, China). The monoclonal antibody to transforming growth factor (TGF) β1 and the rabbit polyclonal antibody to collagen IV were from Abcam (Cambridge, UK). The monoclonal antibody to green fluorescent protein (GFP) was from Cell Signaling Technology (Danvers, MA). The monoclonal antibody to proliferating cell nuclear antigen (PCNA) was from Santa Cruz Biotechnology (Santa Cruz, CA). Peroxidase-conjugated goat anti-mouse IgG and goat anti-rabbit IgG were from Jackson ImmunoResearch (West Grove, PA).

### Cell culture and plasmids

Mesangial cells were prepared from the kidney cortex of male Sprague–Dawley rats as described [21]. Cells maintained in Dulbecco’s modified Eagle medium supplemented with 10% (v/v) fetal bovine serum at 37°C in a humidified 5% (v/v) CO_2_ atmosphere were used between passages 6 and 10. Plasmid pcDNA3.1A-DCN made in our laboratory [22] and plasmid pEGFP (Clontech, Mountain View, CA) were amplified in *Escherichia coli* and purified by the Hispeed Plasmid Midi Kit (QIAGEN) according to the manufacturer’s protocol.

### Preparation of the PEI–pDNA nanocomplex

Branched 25 kDa PEI and pDNA were each dissolved in phosphate-buffered saline (PBS, pH 7.4). Equal volumes of the PEI and pDNA solutions were mixed together to give PEI nitrogen to pDNA phosphate (N/P) ratios of 1, 5, 10, 15 and 20, shaken for 30 s and then kept at room temperature for 30 min. The diameter and zeta potential of the complexes were determined by a laser particle size analyzer (Nicomp™ 380 PSS, CA).

### Transfection of PEI–pEGFP or PEI–DCN into MC

pEGFP was used as a reporter gene to monitor gene expression. The preparation of PEI**–**pEGFP complexes at various N/P ratios is described in the preceding section. At 24 h before transfection, mesangial cells were seeded into 6-well plates at a density of 3 × 10^5^ cells/well. At the time of transfection, the cells were washed twice with PBS, 200 μl mixture solution containing 4 μg PEI**–**pEGFP complex was added to each well followed by incubation for 6 h at 37°C, the transfection agent then was replaced by fresh DMEM containing 10% (v/v) fetal bovine serum and the cells were incubated for 48 h at 37°C. Transfection of pEGFP with Lipofectamine™ 2000 (Invitrogen, Carlsbad) prepared according to the manufacturer’s protocol was used as a positive control. At 48 h following transfection, expression of GFP in transfected cells was observed by inverted fluorescence microscopy. Cells were harvested and used to quantify the transfection efficiency of PEI by flow cytometry (FACScan, BD Biosciences, MD, USA). All transfection experiments were done in triplicate.

MC were grown as described in the preceding section and treated with PEI**–**pDCN complex at an N/P ratio of 20. At 3 days after transfection, geneticin (G418, Sigma) at a concentration of 0.6 mg/ml was added to the cells. Three weeks later, positive clones were detected by Western blotting after culture expansion.

### Cytotoxicity

MC cells were seeded in 96-well plates at a density of 8 × 10^3^ cells/well before transfection and incubation for 24 h at 37°C. The cells were transfected by PEI**–**pEGFP at different N/P ratios as described above and incubated for another 48 h at 37°C. Cells treated with naked plasmid were used as a negative control. The cells were transfected with Lipofectamine™ 2000 as a positive control. Cytotoxicity was assessed using the Cell Counting Kit8 (Dojindo, Tokyo, Japan) according to the manufacturer’s instructions. Relative cell viability was calculated as:

$$\text{Cell viability}(\%)={\text{OD}}_{450(\text{sample})}/OD_{450(\text{control})}\times 100$$

### Western blotting

Cells were harvested in lysis buffer (20 mM Tris at pH 8.0, 150 mM NaCl, 1% (v/v) Nonidet P-40, 1 mM phenylmethylsulfonyl fluoride (PMSF), 1 mM Na_3_VO_4_, 1 mM NaF and 1 μg/ml each of aprotinin, leupeptin and pepstatin) on ice. Proteins (700 μg) of were separated by SDS-PAGE (10% (w/v) polyacrylamide gel) and transferred onto a polyvinylidene fluoride membrane. After blocking with 5% non-fat milk, the membrane was probed with the appropriate primary antibody overnight on a shaker at 4°C and proteins were detected by enzyme-linked chemiluminescence using an ECL kit (Thermo Scientific, Rockford, IL).

### Cell cycle

Analysis of the cell cycle was done by flow cytometry. MC were digested from culture plates by 0.05% (w/v) trypsin and treated with citrate buffer for 30 min, then incubated with RNase for a few minutes followed by addition of propidium iodide for 15 min. The results were analyzed by CellQuest software (BD Bioscience, Mississauga, Ontario, Canada) at the Institute of Biochemistry and Cell Biology, Chinese Academy of Sciences.

### In vivo experimental design

A rat anti-Thy1.1 glomerulonephritis model was made by injection of rabbit anti-rat thymocyte serum (ATS) into the tail vein of male Sprague–Dawley rats as described [23]. At day 3, the rats were anesthetized by an intraperitoneal injection of 10% (w/v) chloral hydrate (0.4 ml/100 g body weight). The left kidney was exposed through a left flank incision, the renal artery was separated and blood flow was interrupted by vessel clamping. MC with PEI–PEGFP or PEI–DCN were trypsinized, suspended in 800 μl of PBS with cell concentration 1 × 10^6^, and injected into the left renal artery using a 29-gauge needle at a rate of 70 μl/s. The cells remained in the left kidney for 5 min and then the blood was allowed to re-perfuse. For the control group, normal mesangial cells were injected into the left renal artery. After 1, 3 and 5 days, 10 rats from the control group and 10 rats from each time group of the experimental group were sacrificed, and both left and right kidneys from each rat were removed separately.

### Immunohistochemistry

Paraffin-embedded (5 μm thick) sections were deparaffinized and endogenous peroxidase was quenched with 3% (v/v) H_2_O_2_. The antigen retrieval was done by microwave at 600 power 10 min, in 0.1 M citric acid-sodium citrate buffer, pH 6.0. Non-specific binding was blocked using 5% (v/v) normal sheep serum for 30 min at 37°C. Sections were then incubated with primary antibodies (anti-EGFP 1:100, anti-DCN 1:200, anti-vimentin 1:50, anti-PCNA 1:100, anti-collagen IV 1:50) at 4°C overnight. Immobilized antibodies were detected by the avidin/biotin/peroxidase technique (Vectastain ABC Kits, Vector Laboratories, UK). Diaminobenzidine was used as the chromogen and hematoxylin was used as the nuclear counterstain. The primary antibody was replaced by PBS in the negative control.

### Statistical analysis

We selected five different glomeruli from each slide at random for the evaluation of staining results. The positive area of each glomerulus was measured by computer-assisted image analysis (KS400, Zeiss, Germany). All values are expressed as mean ± SD. Results were analyzed by Student’s *t*-test with statistically significant difference set at *P* ≤0.05.

## Results and discussion

**Figure 1:**
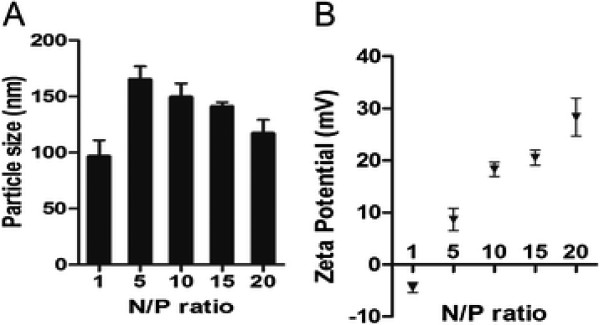
** Particle size (A) and zeta potential (B) of PEI–pDNA complexes at different N/P ratios.** Data are expressed as the mean and standard error of at least three separate measurements.

**Figure 2:**
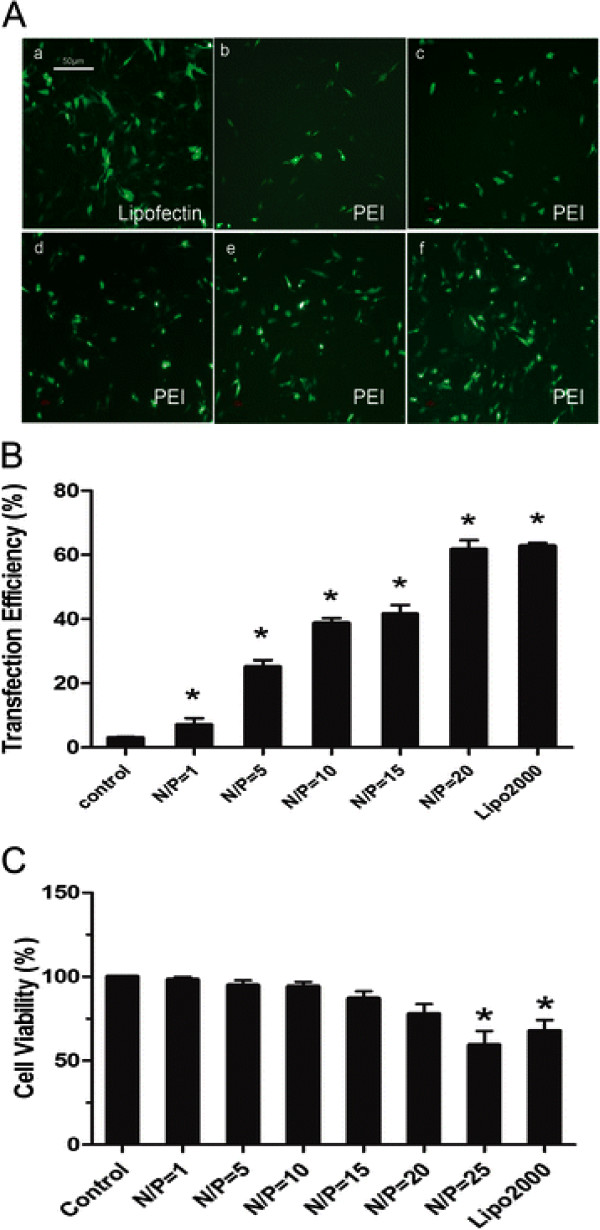
** Evaluation of the transfection efficiency in MC with PEI–pEGFP complexes.A**, Fluorescence micrograph of MC transfected with PEI–pEGFP complexes for 48 h at different N/P ratios. (a) MC were transfected with Lipofectamine™ 2000 as a positive control. (b) N/P 1, (c) N/P 5, (d) N/P 10, (e) N/P 15 and (f) N/P 20. (×100) **B**, The transfection efficiency of MC with different N/P ratios was evaluated by flow cytometry. Cells treated with naked plasmid were used as a negative control. **C**, Cytotoxicity analysis of MC with different N/P ratios by the MTT test. Cells treated with naked plasmid were used as a negative control and cells transfected with Lipofectamine™ 2000 were used as a positive control. *P <0.01 vs control. The results are representative of at least three similar experiments.

It has been reported that the N/P ratio can influence the efficiency of nanoparticle transfection in vitro [24]. To identify the optimal transfection conditions, pEGFP was used as a reporter and PEI**–**pEGFP nanocomplexes with different N/P ratios were prepared. The particle size and the zeta potential of PEI**–**pEGFP nanocomplexes were examined (Figure 1). We found the diameter of the nanocomplex increased from N/P 1 to N/P 5 and then decreased from N/P 5 to N/P 20, while the zeta potential of nanocomplexes indicated positive charges increased with increasing N/P ratio, which is consistent with the report by Ogris [24]. Quantitative analysis of transfection was done with an inverted fluorescence microscope after 48 h. The results showed that transfection efficiency increased with N/P ratios from 1**–**20 and peaked at 20 with a smaller diameter, the expression of EGFP fluorescence was highest and nearly equal to that achieved by Lipofectamine™-mediated gene delivery (Figure 2A). We used flow cytometry to further estimate the transfection efficiency and the result was the same as that described above (Figure 2B). Moreover, the 3-(4,5-dimethyl-2-yl)-2,5-diphenyltetrazolium bromide (MTT) test showed that cell viability was a little lower in cell groups of N/P 1**–**20, but there was no significant difference in cell viability between the control cells and each group of transfected cells with N/P 1 **–** 20. However, cell viability was decreased significantly in the N/P 25 group. The group of Lipofectamine™-transfecting cell also had significant cytotoxicity (Figure 2C). The PEI**–**pDNA complex at an N/P ratio of 20 has the highest transfection efficiency and the lowest cytotoxicity in cultured MC. 

The deliverer for gene therapy generally has two forms, viral and nonviral. Currently, PEI has emerged as a potent nonviral vector for gene therapy in vitro and in vivo. The vehicle with efficient DNA condensation has been used successfully to transfer genes into living cells [25-27]. It has been shown that the N/P ratio influences the efficiency of the gene delivery system and cytotoxicity dramatically, and the optimal N/P ratio differs between cell lines [28,29]. Therefore, it is important to select an optimal N/P ratio of a PEI nanoparticle. In this study, the PEI–pEGFP complexes showed the highest transfection efficiency and relatively lower cytotoxicity at an N/P ratio of 20.

**Figure 3:**
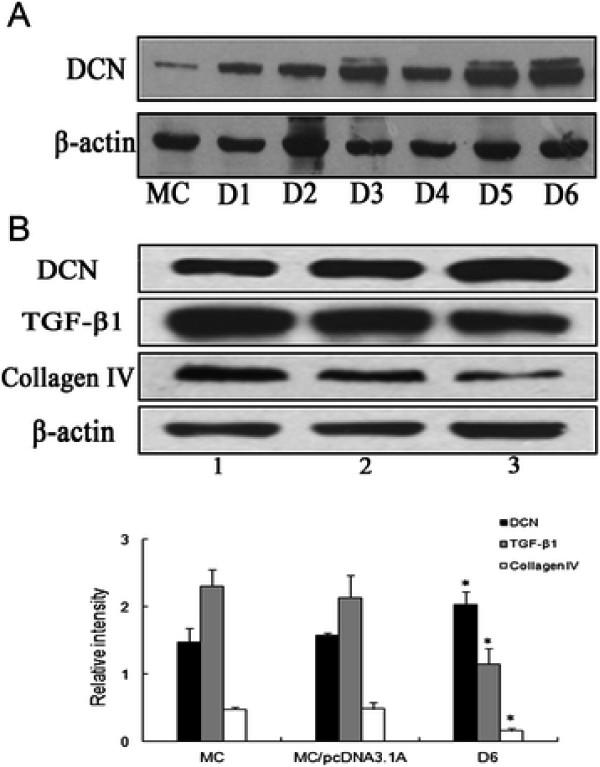
** DCN over-expression inhibited expression of TGF-β1 and collagen IV in MC.A**, The protein level of DCN was assessed by Western blotting in PEI**–**pDCN transfected MC. Normal mesangial cells were used as a control. D1–D6 are different cell clones. **B**, The relative levels of DCN, TGF-β1 and collagen IV protein were determined by Western blotting in D6. Lanes 1, normal mesangial cells; 2, pcDNA3.1A-transfected MC; and 3, DCN-transfected MC (D6). Data are presented as mean ± SD. *P <0.01 vs MC and MC/pcDNA3.1A. The experiment was done at least three times.

To investigate the effect of PEI**–**pDCN on MC biology, we transfected a PEI**–**pDCN nanocomplex into cultured rat MC at N/P 20, which led to over-expression of DCN in MC (Figure 3A). Western blotting (Figure 3B) showed that the protein level of DCN in D6 increased significantly, whereas the expression of TGF-β1 and collagen IV decreased markedly compared to the control cells. The results suggest that over-expression of DCN could inhibit the expression of TGF-β1 and collagen IV of MC in vitro.

To further examine whether over-expression of DCN affects the growth of MC, cell cycle analysis of D6 cells and control cells was done by flow cytometry and the results showed that D6 cells in the G_0_/G_1_ and S phases were 84.47 ± 1.05% and 5.26 ± 0.51%, respectively. Compared to the controls, the difference was significant (Table 1). These data indicate that over-expression of DCN could suppress cell growth by arresting MC in the G_0_/G_1_ phase.

**Table 1:** Analysis of cell cycle in transfected MC by Flow Cytometry

**Groups**	**G**_**0**_**/G**_**1**_**(%)**	**G**_**2**_**/M (%)**	**S (%)**
MC	74.61 ± 3.76	12.06 ± 0.92	13.32 ± 2.03
MC/pcDNA3.1A	75.48 ± 4.17	12.14 ± 1.43	12.37 ± 2.1
D6	84.47 ± 1.05*	10.53 ± 2.79	5.26 ± 0.51*

*P < 0.05 vs MCs and MC/pcDNA3.1A.

**Figure 4:**
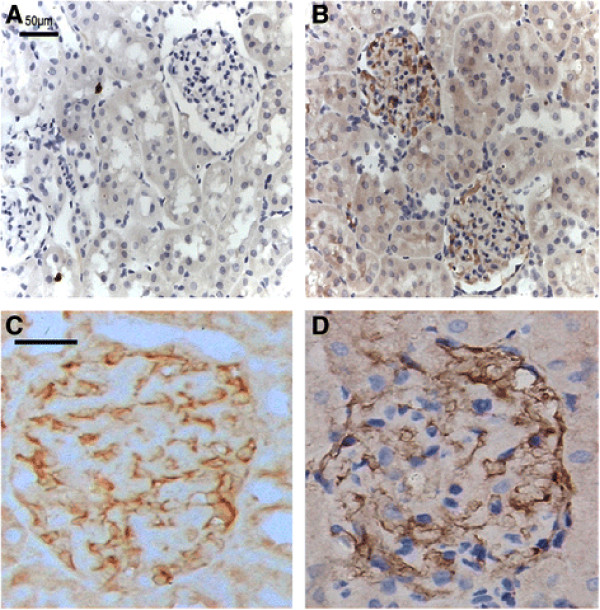
** Location of PEI–pEGFP or PEI–DCN in injected glomeruli.** At 48 h after injection of the MC vector with PEI**–**pEGFP or PEI**–**DCN, paraffin sections of kidney were prepared and examined by immunohistochemistry. (**A**) Normal control glomerulus negative for EGFP staining. (**B**) Glomerulus of injected kidney with PEI**–**pEGFP expressing MC, the results show EGFP-positive cells are located mainly in the mesangial area and a few epithelial cells of the proximal tubule. (a,b ABC, ×100) (**C**) Glomerulus of injected kidney with PEI**–**DCN expressing MC. The exogenous DCN-positive cells have the same localization as EGFP in glomeruli. (**D**) Glomerulus of injected kidney with PEI**–**DCN expressing MC. The vimentin-positive MC are distributed mainly in the mesangium of glomeruli, which is consistent with the distribution pattern of PEI-DCN positive cells (c,d ABC × 200).

We used pEGFP as a reporter gene and prepared stably transfected MC with PEI**–**pEGFP complexes to estimate the glomerular targeting of the MC vector. We injected MC vector into the normal rat kidney via the left renal artery and the rats were sacrificed after 2 days. Immunohistochemistry showed that about 65 ± 2.13% of the glomeruli in the left kidney were positive for EGFP staining and these cells were located mainly in the mesangium and the glomerular capillary (Figure 4B), whereas the right kidney of the same animal was negative for EGFP and served as the control (Figure 4A). Subsequently, the MC vector with PEI**–**DCN was also injected into the normal rats kidney. Immunohistochemical staining for exogenous DCN expressing cells had the same localization in glomeruli as EGFP (Figure 4C), which is consistent with the distribution pattern of vimentin-positive cells that is recognized as a mesangial cell marker in glomeruli (Figure 4D) [30]. By contrast, DCN staining was negative in the normal kidney where DCN was expressed at very low or even undetectable levels. 

The rat anti-Thy1.1 glomerulonephritis model was induced by injection of rabbit anti-thymocyte serum into tail veins. The proliferation of MC and matrix in mesangium occurred 3 days after injection and more severe changes were seen at day 7.

**Figure 5:**
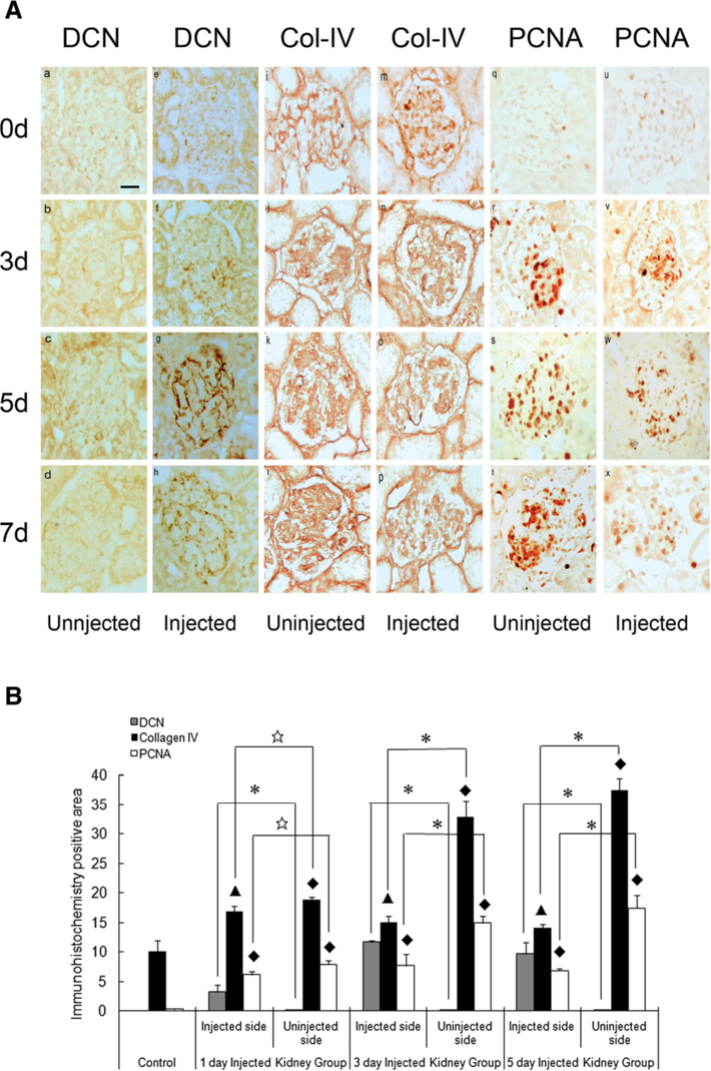
** Immunohistochemistry results of DCN, collage IV and PCNA in rat Thy1.1 glomerulonephritis.** Immunostaining of (**A**) DCN, (**B**)collage IV and (**C**) PCNA in rat Thy1.1 glomerulonephritis. (a–d) DCN expression in glomerulus of uninjected kidney. (e–h) DCN-positive distribution in glomerulus 1, 3 and 5 days after injection with PEI**–**DCN. Level of collagen IV expression in glomerulus of (i–l) uninjected kidney and (m–p) injected kidney. PCNA protein level in glomerulus of (q–t) uninjected kidney and (u–x) injected kidney. (ABC × 200). B, Immunohistochemistry results and statistical analysis. ⋆, *P* <0.05 vs uninjected side; *, *P* <0.01 vs uninjected side; ▴, *P* <0.05 vs control; ♦, *P* <0.01 vs control.

Furthermore, the rat models were treated by MC (D6) vector with PEI**–**pDCN via injection of the left renal artery. Immunohistochemistry showed the DCN expression of D6 cells was located in glomeruli 1 day after the injection; its expression level was enhanced at day 3 and decreased slightly at day 5. However, the expression of DCN in all groups was significantly higher than that in the uninjected (right) kidney; DCN expression was totally absent from the uninjected control kidneys (Figure 5A and B).

The expression of collagen type IV and PCNA in the glomeruli was detected by immunohistochemical staining and the results showed that the expression levels of both collagen type IV and PCNA in glomeruli in the PEI**–**pDCN group were decreased significantly compared to that in uninjected kidneys (Figure 5A and B). Thus, matrix synthesis and cell proliferation were inhibited effectively by DCN over-expression in D6 cells.

Efficient targeted gene therapy in vivo is still a major challenge. PEI is one of the most efficient nonviral vectors but there are still many problems limiting its systemic application following intravenous administration in vivo [31]. For example, the PEI–DNA complex is restricted by a shortage of organ targeting; it is usually expressed and aggregates in lung tissue via rat tail vein injection [32,33]. Cationic polymers and DNA are known to bind serum proteins, which might serve to opsonize the complexes and perhaps promote rapid clearance by macrophages in the bloodstream [34-36]. Therefore, the recent trial of PEI delivery of genes to target cells in vivo were done mainly by direct injection either into the target tissue [13,14,37] or into its blood supply [38,39] or by introduction with an appropriate cell vector [20].

PEI-mediated gene delivery to rat kidney was reported by Boletta et al. [38]. The PEI**–**DNA particles injected into the left kidney via the left renal artery were localized exclusively in the proximal tubule, suggesting that direct injection with PEI**–**DNA complexes in kidney could be an effective route for renal tubulointerstitial injury instead of glomerular diseases. Furthermore, the ex vivo trial using a mesangial cell as a vector to transfer PEI**–**DNA is more successful for specific targeting of glomeruli [20]. With the homing instinct of a cell vector, MC with β-galactosidase plasmid injected into the left kidney via the renal artery were located and enzyme expressed mainly in the glomeruli capillary and mesangium. This is a good example of attractive targeting strategy for transgene therapeutics of glomerular diseases. In this study, we transferred the MC vector with the PEI**–**DCN complex into the kidney of rat anti-Thy1.1 nephritis. The results showed that the exogenous MC with DCN expressing were located mainly in the mesangium and the glomerular capillary. Rat anti-Thy1.1 nephritis models are characterized by MC proliferation accompanied with ECM deposition. Over-expression of DCN in diseased glomeruli by injection of MC with a PEI**–**DCN complex could alleviate the pathological changes of rat nephritis. Thus, the DCN gene delivered by a PEI**–**DNA nanocomplex with the MC vector is proved to be a promising therapeutic reagent for the treatment of glomerulonephritis.

## Conclusions

This study demonstrated that PEI-mediated DCN gene transfer to MC is effective and could be used as a gene-stable transfection carrier in vitro. We transferred a PEI–DCN complex into the glomeruli of a rat anti-Thy1.1 nephritis model by the MC vector with the PEI–DCN complex by injection into the left renal artery. We observed expression of the DCN protein in transfected MC in injected glomeruli, which have an important antagonistic effect on glomerular lesion of rat anti-Thy1.1 nephritis. We have shown the ex vivo gene transfer of a PEI–DCN complex to glomeruli is feasible and could be a useful method for gene therapy of glomerular diseases.

## Abbreviations

PEI: polyethylenimine; DCN: decorin; MC: mesangial cell; pDNA: plasmid DNA; HVJ: hemagglutinating virus of Japan; PBS: phosphate-buffered saline; GFP: green fluorescent protein; PMSF: phenylmethylsulfonyl fluoride; ATS: anti-rat thymocyte serum.

## Competing interests

The authors declare that they have no competing interest.

## Authors’ contributions

JYS performed the experiments and drafted the manuscript. YS, HJW and HXZ carried out the partial experiments and participated in the mechanism analysis. ZHZ and QC helped in the technical support for the experiments. ZGZ designed the experiments and revised the manuscript. All authors read and approved the final manuscript.
